# Development of a Curcumin-Loaded Polymeric Microparticulate Oral Drug Delivery System for Colon Targeting by Quality-by-Design Approach

**DOI:** 10.3390/pharmaceutics12111027

**Published:** 2020-10-27

**Authors:** Dana Hales, Lucia Ruxandra Tefas, Ioan Tomuță, Cristian Moldovan, Diana Gulei, Raluca Munteanu, Alina Porfire

**Affiliations:** 1 Department of Pharmaceutical Technology and Biopharmacy, Faculty of Pharmacy, University of Medicine and Pharmacy “Iuliu Haţieganu”, 41 Victor Babeș Street, 400012 Cluj-Napoca, Romania; dudas.dana@umfcluj.ro (D.H.); tefas.lucia@umfcluj.ro (L.R.T.); aporfire@umfcluj.ro (A.P.); 2 Research Center for Advanced Medicine—MedFUTURE, University of Medicine and Pharmacy “Iuliu Haţieganu”, 4-6 L. Pasteur Street, 400349 Cluj-Napoca, Romania; moldovan.cristian@umfcluj.ro (C.M.); diana.gulei@umfcluj.ro (D.G.); raluca.muresan@umfcluj.ro (R.M.)

**Keywords:** curcumin, Eudragit^®^ FS, polycaprolactone, microspheres, colon-specific delivery, quality-by-design

## Abstract

The purpose of this study was to apply the quality-by-design (QbD) approach for the development of colon-targeted curcumin-loaded polymeric microparticles (Col-CUR-MPs). The proportion of the enterosoluble polymer (Eudragit^®^ FS) in the polymeric matrix, curcumin concentration, and the concentration of the polymer mixture (Eudragit^®^ FS-polycaprolactone) were identified as potential risk factors for the quality of the final product following risk assessment. The influence of these variables on the critical quality attributes (CQAs) of Col-CUR-MPs was investigated. Therefore, a central composite face experimental design was used in order to determine the functional relationships between variables and product CQAs. The obtained regression model and contour plots were used to establish the design space. Finally, the model was validated by preparing two microparticulate formulations, one corresponding to the robust setpoint from within the design space and one outside the established design space, and calculating the percentage bias between the experimental and predicted values. The in vivo study, which was conducted on a fluorescein-loaded formulation that corresponded to the robust setpoint determined by QbD and that contained a mixture of polycaprolactone and Eudragit^®^ FS (60:40, *w/w*), confirmed the colon-targeting qualities of this formulation.

## 1. Introduction

Inflammatory bowel disease (IBD) is a chronic, relapsing, and debilitating inflammatory disorder of the gastrointestinal tract (GIT), presenting two major forms: ulcerative colitis (UC) and Crohn’s disease (CD). The difference between UC and CD is the fact that in UC, the inflammation is limited to the colon, while CD affects any part of the GIT, commonly the terminal ileum or the perianal region [1]. There are millions of individuals worldwide affected by IBD that have to take medication throughout their whole life, as there is no permanent cure for this disease [2]. Anti-inflammatory drugs (e.g., corticosteroids), aminosalicylates, immunosuppressants, antibiotics and targeted therapies (e.g., anti-TNF-α monoclonal antibodies) are mainly used in the treatment of IBD. The drawback of these drugs is the fact that they cause serious side effects such as osteoporosis, acute pancreatitis and infection, and diarrhea, the reason being their non-specific delivery at the site of inflammation and the long-term treatment period. Therefore, the development of alternative agents with high therapeutic efficacy and low side effects on the one hand, and of a delivery system able to selectively target the inflamed colonic tissue on the other hand, are of critical importance [1,2].

Curcumin is a natural herbal polyphenolic product, extracted from the turmeric pigment of *Curcuma longa*, which has been used for decades for its pleiotropic effects: anti-inflammatory, antioxidant, anti-carcinogenic, antimicrobial, hepatoprotective, anti-hyperlipidemic, and anti-angiogenic. The anti-inflammatory effect of curcumin has already been demonstrated in various diseases such as Alzheimer’s disease, Parkinson’s disease, multiple sclerosis, epilepsy, and cerebral injury. The anti-inflammatory effect has also been reported in IBD, as curcumin is able to regulate the oxidant/anti-oxidant balance and modulate the release of inflammatory mediators, namely TNF-α and nitric oxide [1]. Another important effect of curcumin is its capacity to inhibit tumor cells’ proliferation, to hinder the activation of the nuclear factor-kappa B and to reduce multiple drug resistance through the down regulation of P-glycoprotein expression [3]. Regarding the safety of this natural drug, clinical trials have shown that curcumin is relatively safe for humans, and as a result, it has been acknowledged as a “generally regarded as safe” (GRAS) compound by the United States Food and Drug Administration (FDA) [4].

In spite of its numerous advantages, curcumin presents several limiting factors such as low solubility in water (i.e., 0.4 mg/mL at pH 7.3), instability, and rapid decomposition at neutral and alkaline pH values and instability towards light. Due to its hydrophobic character, curcumin shows low absorption, poor bioavailability by oral route, high intestinal metabolic rate, and rapid elimination from the body. Numerous approaches have been made to improve the bioavailability of curcumin and overcome the physicochemical limitations. The main strategies are based on loading the compound in micro- and nanocarriers (e.g., pellets, polymeric microparticles, liposomes, polymeric nanoparticles, or micelles) which are able to deliver curcumin to colitis tissue and improve its solubility, protect it from degradation, and facilitate its delivery by specifically targeting the inflamed colon [1,2,3]. The development of curcumin-loaded micro- and nanocarriers is used not only in colon-specific drug delivery systems, but in all the delivery systems pursuing the improvement of curcumin bioavailability, drug loading, stability, and programmed release [5,6].

Orally administered microparticulate carriers have been recognized as a promising drug delivery system for IBD therapy due to their high drug loading, sustained drug release capacity, and colitis tissue-targeting ability [2]. The oral route has been considered the most effective approach to IBD therapy due to various advantages such as high patient compliance and cost-effectiveness. Various polymeric microparticulate approaches are used for targeting drugs to the colon for the treatment of UC such as pH-dependent, time-dependent, microflora- or enzyme-activated systems, mucoadhesive, and pressure-controlled-based systems. Microparticles should be biocompatible and biodegradable and have the ability to deliver drugs specifically to the confined area of the GIT [7]. There is a great variety of curcumin-loaded colon-specific drug delivery systems presented in published data, such as liquisolid tablets [8], *Bacillus* spore-based oral carriers [9], or nanoparticles [10,11,12,13]. However, there are only a few studies that have developed microparticulate systems for the specific delivery of curcumin to the colonic tissue: porous poly(lactic acid/glycolic acid) (PLGA) microparticles [14], bowl-shaped PLGA microparticles [2], or pH-sensitive Eudragit^®^ S100/PLGA microparticles. Regarding drug administration via the oral route in IBD therapy, microparticles may target colitis tissue based on the epithelial enhanced permeation and retention effect. This effect was associated with the disturbances of the UC colonic tissues which were observed by histopathological examination, namely the disruption of the intestinal barrier function, high permeability of the epithelium, and inflammatory cells’ accumulation into the mucosa [4,14]. pH-sensitive systems are the most widely used delivery systems in IBD [4]. These systems are based on pH-dependent (enteric) polymers, mainly cellulose acetate phthalate, hydroxypropylmethylcellulose phthalate or derivatives of methacrylic acid (Eudragit^®^ S 100, Eudragit^®^ L, Eudragit^®^ FS, Eudragit^®^ P4135 F) [4,7,15]. Eudragit^®^ FS is an anionic copolymer based on methyl acrylate, methyl methacrylate and methacrylic acid, which dissolves above pH 7.0 by salt formation, allowing targeted colon delivery [16]. To obtain a more sustained drug release, the biodegradable and hydrophobic polyester—polycaprolactone can be introduced into the system. There is no published data on the use of the two polymers mentioned above for the encapsulation of curcumin in microparticulate systems.

Quality-by-design (QbD) is a systematic approach of the design and development of manufacturing processes which focuses on understanding and controlling the pharmaceutical quality of drugs and the formulation and production variables in order to ensure a pre-determined quality for the final product [17,18]. The ICH Quality guidelines, especially Q8 (R2) Pharmaceutical Development [19], Q9 Quality Risk Management [20], Q10 Pharmaceutical Quality System [21], and Q11 Development and Manufacture of Drug Substances [22], are the basic guidelines for the pharmaceutical industry and are recommended for adoption to the regulatory bodies of the European Union (European Medicines Agency (EMA)), Japan (Pharmaceuticals and Medical Devices Agency (PMDA)), and USA (FDA) [17]. According to ICH Q8 (R2) and literature data, pharmaceutical development should include the following elements: (1) defining the quality target product profile (QTPP); (2) identifying potential critical quality attributes (CQAs) of the drug product and critical process parameters (CPPs); (3) identifying, through prior knowledge, experimentation, and risk assessment, the material attributes and process parameters that can have an effect on product CQAs; (4) determining the functional relationships that link material attributes and process parameters to product CQAs through the development of multivariate experiments using Design of Experiments (DoE); (5) establishing a design space, and (6) ensuring continual improvement and innovation throughout the product lifecycle [19,23].

Herein, we described the first attempt to fabricate optimal colon-targeted curcumin-loaded polymeric microparticles (Col-CUR-MPs) using the polymeric combination Eudragit^®^ FS-polycaprolactone. Therefore, we have focused on the systematic optimization of the basic composition of Col-CUR-MPs by the QbD approach, with the aim to protect curcumin from degradation in gastrointestinal fluids and to allow curcumin release at the site of the inflamed colitis tissue. After defining the desired characteristics of the microspheres for colon-specific delivery and establishing the potential CQAs, we identified CPPs through risk assessment. Col-CUR-MPs were prepared by an emulsion solvent evaporation technique using a central composite face experimental design approach to optimize the composition of the microspheres. Further on, the impact of formulation variables on the CQAs was studied using multivariate DoE tools, the design space was established, and finally, the model was tested for accuracy and robustness. The characteristics of the optimum formulation were appropriate for colon targeting, as the microspheres presented high drug encapsulation and gradual drug release. Moreover, in order to determine the colonic drug delivery system’s effectiveness in the specific targeting of the colon, the microspheres were evaluated by an in vivo imaging system (IVIS).

## 2. Materials and Methods

### 2.1. Materials

Curcumin, polycaprolactone (average mol *wt* 45,000), poly(vinyl alcohol) (87–90% hydrolyzed, average mol *wt* 30,000–70,000), and fluorescein isothiocyanate (FITC) were purchased from Sigma-Aldrich (St Louis, MO, USA). Eudragit^®^ FS 100 was a kind gift from Evonik Nutrition&Care GmbH (Darmstadt, Germany). Dichloromethane, dimethyl sulfoxide, and acetonitrile were purchased from Merck KGaA (Darmstadt, Germany). Ethyl acetate was purchased from Chimopar (Bucharest, Romania). Potassium dihydrogen phosphate and sodium hydroxide were purchased from Chemical Company (Iași, Romania). Sodium chloride was purchased from Lach-Ner (Brno, Czech Republic). All other chemicals were of analytical grade.

### 2.2. Preparation of Col-CUR-MPs

The microspheres were prepared by an oil-in-water emulsion method followed by solvent evaporation, as previously described by Anchi et al., with some modifications [24]. Briefly, curcumin was dissolved in 2.5 mL dimethyl sulfoxide, Eudragit^®^ FS 100 was dissolved in 2 mL ethyl acetate, and polycaprolactone was dissolved in 2 mL dichloromethane. After combining the three solutions, the organic phase was added to 20 mL aqueous solution of 2% poly(vinyl alcohol) and stirred with a magnetic stirrer for 2 min at 340 rpm to obtain the oil-in-water emulsion. This emulsion was added to 100 mL double-distilled water and stirred with a magnetic stirrer for 24 h at 510 rpm, at room temperature, in order to remove the organic solvents from the internal phase. During the solvents’ evaporation, the polymers precipitated and the microparticle cores solidified. The microspheres were collected by filtration (nylon membrane, 0.45 μm) and dried at room temperature.

The FITC-loaded microspheres evaluated by the in vivo imaging system (IVIS) were prepared by the same technique as described above. In order to obtain stronger fluorescent microspheres, curcumin was replaced by FITC. Two formulations were prepared: one containing 100% polycaprolactone (corresponding to the reference formulation) and one containing a mixture of polycaprolactone and Eudragit^®^ FS (60:40, *w/w*) (corresponding to the test formulation, which had the same polymer ratio as the robust setpoint formulation established by the QbD approach).

### 2.3. Physicochemical Characterization of Col-CUR-MPs

The dried microspheres were characterized in terms of particle size, particle size distribution, curcumin entrapment efficiency and drug loading, yield, and the amount of curcumin released at different time intervals during 24 h.

#### 2.3.1. Particle Size Analysis and Particle Size Distribution

The particle size (expressed in μm) and particle size distribution (expressed in %, representing the relative standard deviation) of Col-CUR-MPs were determined by laser light diffraction, using the Analysette 22 MicroTec plus apparatus (Fritsch, Weimar, Germany) equipped with a dry dispersion unit module.

#### 2.3.2. Determination of Curcumin Entrapment Efficiency and Drug Loading

The amount of curcumin entrapped in the Col-CUR-MPs was determined by high-performance liquid chromatography (HPLC) analysis with fluorescence detection. In short, the samples were dissolved in acetonitrile, and the resulting solution was further diluted with a mixture of acetonitrile and 0.2% formic acid solution (40:60, *v/v*). The precipitate was separated by centrifugation at 15,000 rpm for 10 min, and 5 μL of clear sample were injected into the HPLC system. The chromatographic separation was conducted on an Agilent 1100 Series HPLC system (Agilent Technologies, Santa Clara, CA, USA) equipped with a Zorbax C18 column (3.5 μm) and a fluorescence detector. The mobile phase was composed of acetonitrile and 0.2% formic acid (40:60, *v/v*) and had a flow rate of 1 mL/min. An isocratic elution was used. The excitation and emission wavelengths were set at 425 and 533 nm [25]. All measurements were performed at 30°C. The entrapment efficiency was calculated according to the following equation:

Entrapment efficiency (%) = (Amount of drug entrapped in the microspheres/Amount of drug added in the microspheres) × 100(1)

and the drug loading (DL) was calculated according to the equation presented below:

DL (%) = (Amount of drug entrapped in the microspheres/Total weight of the microspheres) × 100(2)

#### 2.3.3. Percentage Yield

The percentage yield was analyzed as a factor, as it is economically important to know the conditions in which the product is obtained with the best possible yield. The dried microspheres were weighed and the percentage yield of the prepared microspheres was calculated using the following formula:

Yield (%) = (Actual weight of the microspheres/Total weight of all non-volatile components) × 100(3)

#### 2.3.4. In Vitro Drug Release Study

Curcumin release from the Col-CUR-MPs was assessed through dissolution testing at a temperature of 37 °C and a rotation speed of 50 rpm, using a volume of 20 mL dissolution medium in order to provide sink conditions. The microspheres (5 mg) were suspended in 20 mL 0.05% Tween 80 in HCl 0.1 M (pH 1.2) to simulate the gastric environment for 2 h. After 2 h, the microspheres were separated by centrifugation (15,000 rpm, 15 min) and further suspended in 0.05% Tween 80 in phosphate buffer 0.05 M (pH 6.8) to simulate the intestinal environment for 22 h. Samples of the dissolution medium (1 mL) were withdrawn at predetermined time intervals (2, 4, 6, 12, and 24 h) and replaced by 1 mL fresh media. The amount of curcumin in the withdrawn samples was assayed by the same HPLC method used to determine the entrapment efficiency of curcumin in the microspheres. The dissolution study was performed in duplicate for each of the 17 formulations and the results are expressed as mean ± standard deviation.

### 2.4. QbD Approach

#### 2.4.1. Identification of QTPP and Determination of CQAs of Col-CUR-MPs by Risk Assessment

The QTPP and the CQAs of Col-CUR-MPs were established based on scientific, regulatory, and practical considerations and limitations. The risk assessment is performed in order to identify the CQAs that have the greatest chance of affecting the final quality of the product or producing product failure. Based on scientific literature information and on preliminary formulation studies, the particle size and particle size distribution, drug loading, drug entrapment efficiency, and percentages of drug released in media simulating the GIT environment were chosen as CQAs of our microspheres for colon-specific delivery. Potential risk factors which are likely to influence the CQAs have been identified through risk analysis; three risk variables were selected to be further studied and were included in a central composite face experimental design.

#### 2.4.2. DoE

Based on the risk analysis results, three key variables were selected as independent factors to be investigated, namely the Eudragit^®^ FS proportion (X_1_), the curcumin concentration (X_2_), and the concentration of the polymer mixture (X_3_). Each independent factor was assigned two levels, low and high, which were represented by the values of −1 and +1, respectively (Table 1). A central composite face experimental design was used to study the influence of the risk factors on the preparation of Col-CUR-MPs. The matrix of the experimental design, which comprised 17 experiments, of which three were replicates (N15, N16, and N17), is presented in Table 2. The design of the study was developed using Modde 12.1 Pro software (Sartorius Stedim Biotech, Malmö, Sweden). The responses (dependent variables) evaluated were the particle size (Y_1_), particle size distribution (Y_2_), curcumin drug loading (Y_3_), curcumin entrapment efficiency (Y_4_), yield (Y_5_), and the amount of curcumin released during 24 h in media that simulated the gastric (Y_6_—percentage of curcumin released after 2 h) and intestinal environment (Y_7_–Y_10_—percentage of curcumin released after 4 h, 6 h, 12 h, and 24 h). The data were fitted by means of partial least squares using the statistical module of Modde 12.1 Pro software. The experiments were performed in a random order to reduce the experimental variability.

#### 2.4.3. Establishment of the Design Space

The design space is the multidimensional combination and interaction of input variables and CPPs that have been demonstrated to provide the assurance of quality, according to ICH Q8 (R2) [19]. In this study, the design space was established with Modde 12.1 Pro software, as the combination of factors for which the target specifications of the Col-CUR-MPs were met at specific risk levels. In order to perform the formulation optimization, the models developed in the experimental design were validated by preparing and determining the CQAs of one formulation within the design space (robust setpoint) and one formulation outside the design space. The formulations were characterized in terms of the CQAs mentioned before. The experimental results were compared to the predicted ones, for the investigated responses, and the percentage bias was calculated [25].

### 2.5. In Vivo Release Imaging in Mice

The FITC-loaded microspheres were prepared by the method described in Section 2.2, and the in vivo release testing was performed as previously described by Chen et al., with some modifications [26]. Eight-week-old female mice purchased from Charles River Laboratories, Inc. (Wilmington, MA, USA) were included in the study. The mice were housed in an IVC2-SM-56-IIL rack system (Acéllabor, Vecsés, Hungary) with individual ventilated cages supplied with HEPA filtered air, at a standard temperature of 22 °C ± 2 °C and a relative humidity of 55% ± 10% in a 12:12 h light:dark cycle with ad libitum access to autoclaved water and pelleted feed. After 24 h fasting, the mice, each weighing 20–23 g, were divided in three cohorts and were administered the following treatments through oral gavage: (1) the negative control group received 200 µL aqueous solution of 1% carboxymethylcellulose sodium salt, (2) the reference group received 4 mg FITC-loaded microparticles containing 100% polycaprolactone suspended in 200 μL aqueous solution of 1% carboxymethylcellulose sodium salt, and (3) the test group received 4 mg FITC-loaded microparticles containing a mixture of polycaprolactone and Eudragit^®^ FS (60:40, *w/w*) suspended in 200 μL aqueous solution of 1% carboxymethylcellulose sodium salt. The polymer composition of the test formulation was the one established by the design space analysis and corresponded to the robust setpoint. The treated mice were immediately anesthetized with isoflurane, and the transit of FITC-loaded microparticles in the GIT and FITC absorption were monitored with IVIS SPECTRUM—IVIS Imaging System (PerkinElmer, Inc. Waltham, MA, USA) at 10, 20, 46, 50, 55, and 140 min after treatment administration. The settings for the image acquisition were: epi-illumination, excitation filter: 465 nm, emission filter: 520 nm, lamp level: high, binning of 8, FOV: 13.2, aperture F4, and exposure time of 1 s. The images were processed using Living Image^®^ 4.5.2 Software (smoothing 3 × 3). All experimental protocols were reviewed and approved by the Ethics Committee of the University of Medicine and Pharmacy “Iuliu Haţieganu” (Decision no. 267/28.07.2020) and were conducted in accordance with the EU Directive 63/2010. The assessment of fluorescence intensity was made with the integrated software mentioned above by measuring the ROI (region of interest) values. Specifically, three anatomical areas were defined using the free drawing option: stomach, small intestine, and large intestine (colon), areas that overlapped across all experimental groups. The obtained median ROI values for each area, average radiant efficiency (p/s/cm^^2^^/sr)/(µW/cm^2^), were calculated by excluding the values obtained for the negative control group (considered background values) from the reference group and the test group, respectively.

## 3. Results and Discussion

### 3.1. Identification of QTPP and CQAs of Col-CUR-MPs by Risk Assessment

In a QbD study, the first step is to establish the desired characteristics of the final product—in other words, to set the goals for the QTPP [27]. Based on the QTPP, defined as a prospective summary of the quality characteristics of a drug product, the CQAs (the characteristics of a drug product that should be within an appropriate limit, range, or distribution to ensure the desired product quality) were defined by means of a risk assessment [19]. In this study, we focused on obtaining curcumin loaded microspheres with the following characteristics: high drug loading and entrapment efficiency, minimum drug release in the gastric simulated environment, and in the first hours spent in intestinal simulated environment and maximum drug release at the end of the 24 h dissolution study. Hence, the particle size and particle size distribution, drug loading, drug entrapment efficiency, and percentages of drug released in media simulating the GIT environment were identified as CQAs of the final product, microspheres for colon-specific delivery. The reason why these characteristics were considered critical for the microspheres for colon-specific delivery was their influence on the ability of the system to target the colonic mucosa and to exert its therapeutic potential.

Risk assessment is a process which implies hazard identification, risk analysis and risk evaluation, its purpose being to identify the attributes which have the highest risk of affecting the CQAs [20]. These high-risk attributes will be further introduced as variables in a design of experiments study in order to establish a product or process design space [27,28]. The study of the literature and preliminary formulation studies allowed the identification of risk factors which have a high potential to influence the CQAs of the Col-CUR-MPs. These factors were classified in formulation factors and process parameters. Process parameters, such as stirring speed and stirring time during the preparation of the oil-in-water emulsion, were kept constant throughout the experimental plan, as the preliminary formulation studies indicated they do not represent risk factors. The formulation factors which were studied through the experimental design were the proportion of the pH-dependent, enterosoluble polymer (Eudragit^®^ FS), the curcumin concentration, and the concentration of the polymer mixture (Eudragit^®^ FS-polycaprolactone).

Particle size and particle size distribution are critical physicochemical properties of microparticles, as these parameters have a direct impact on the stability, biodistribution, and mucoadhesive properties, and determine the in vivo uptake of the colon-specific drug delivery system by inflamed cells/tissues which are present in inflammatory bowel diseases [4]. Literature data state that the pharmacological effect is better in the case of nanoparticulate and microparticulate (below 200 μm) systems for colon targeting, for the reason that the microparticles and submicroparticles are easily taken over by cancerous and inflamed cells or tissues [29]. Moreover, multiparticulate systems have been shown to be uniformly distributed throughout the colon as against to single unit systems, which reduces the irritation and provides a reproducible in vivo drug release [30]. Another aspect concerning the influence of particle size on drug release is that according to Fick’s laws of diffusion, the concentration gradients are expected to decrease, resulting in decreasing drug release rates with increasing microparticle size. However, the porosity of microparticles can also play an important role in addition to the system size; therefore, it is difficult to theoretically predict the influence of particle size on drug release [31]. In a previous study, the increase in the ratio of Eudragit^®^ FS-30D determined a notable size increase of the microspheres prepared based on the combination Eudragit^®^ FS-30D/Eudragit^®^ RS-PO polymers; therefore, the proportion of the pH-dependent polymer (Eudragit^®^ FS) needs further investigation in order to establish if the effect is similar in the case of the polymer mixture Eudragit^®^ FS-polycaprolactone used in this study [32]. There are also data in the literature that showed that the size of microspheres was increased with the increase in the concentration of drug [33]. The concentration of the polymer mixture is also likely to influence the particle size; the increased viscosity of the organic solution when increasing the concentration of the polymer mixture could lead to the increase of particle size; therefore, the optimum concentration should be determined [32].

Obtaining an optimum drug loading and entrapment efficiency is essential for a colon drug delivery system, as a part of the loaded drug is usually released before the system reaches the colon. Furthermore, a higher amount of encapsulated drug will reduce the production costs by preventing the loss of raw materials and will ensure a greater flexibility in dosing by increasing drug concentration in the final formulation [34]. In a previous study, the combination ratio Eudragit^®^ FS-30D/Eudragit^®^ RS-PO influenced the encapsulation efficiency of enoxaparin sodium due to the ionic bonds that formed between the drug and the polymers [27]; therefore, there is a need to investigate whether the proportion of the pH-dependent polymer (Eudragit^®^ FS) affects the curcumin drug loading and entrapment efficiency. Drug concentration has been previously shown to influence drug loading and entrapment efficiency; therefore, this variable was also selected as a formulation factor of the Col-CUR-MPs [35]. Regarding the polymer concentration, a study on huperzine A-PLGA microspheres investigated the influence of formulation factors on the drug encapsulation efficiency and in vitro release, and revealed that a polymer concentration increase in the oil phase improved encapsulation efficiency [36]. Therefore, it is justified to determine the influence of the concentration of the polymer mixture Eudragit^®^ FS-polycaprolactone on the drug loading and entrapment efficiency of curcumin in the Col-CUR-MPs.

Drug release is a critical parameter for colon-specific drug delivery systems: the maximum release in the gastric medium must be lower than 10% [37] and the delivery system must provide a sufficient lag time and release as little drug as possible in the small intestine in order to allow the release of the drug in large amounts at the site of inflammation, in the colon, therefore gaining colon-specific drug delivery ability [38]. The variation of the three formulation factors, namely the proportion of the pH-dependent polymer (Eudragit^®^ FS), the curcumin concentration, and the concentration of the polymer mixture (Eudragit^®^ FS-polycaprolactone), in the experimental plan, ought to show how their variability influences drug release.

After the risk analysis, we investigated the impact of the risk factors on the CQAs of Col-CUR-MPs, by an experimental design.

### 3.2. DoE

Design of experiments can be defined as the strategy for establishing experiments in such a way that the necessary information is obtained as efficiently and accurately as possible. It allows the simultaneous study of different formulation and process variables, establishing the links between them and their influence on experimental responses, by performing a minimum number of experiments [39].

**Figure 1:**
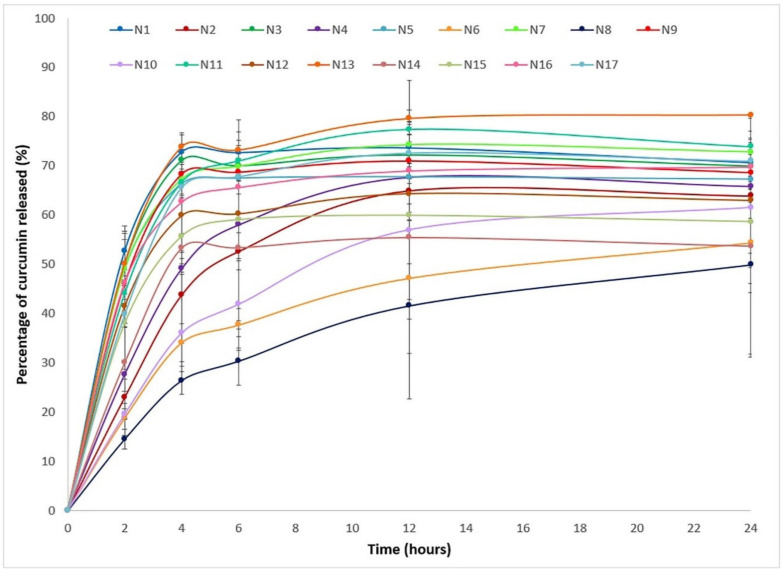
In vitro release profile of curcumin from colon-targeted curcumin-loaded polymeric microparticles (Col-CUR-MPs).

The 17 experiments were carried out according to the experimental design, and the results obtained for responses Y_1_–Y_5_ are given in Table 3, while Figure 1 shows the in vitro release profile of curcumin from Col-CUR-MPs (Y_6_–Y_10_).

### 3.3. Summary of Fit

The validity of the experimental design and the fitting of the regression model to the experimental data may be verified by determining the following statistical parameters: *R*^2^ (“goodness of fit”), *Q*^2^ (“goodness of prediction”), validity, reproducibility, and analysis of variance (ANOVA) [25,39].

**Figure 2:**
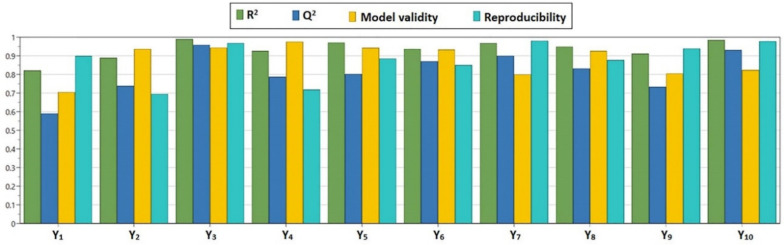
Summary of fit plot showing *R^2^*, *Q^2^*, model validity and reproducibility; *R*^2^—regression coefficient; *Q*^2^—predictive power of the model; Y_1_—particle size (μm); Y_2_—particle size distribution (%); Y_3_—drug loading (%); Y_4_—entrapment efficiency (%); Y_5_—yield (%); Y_6_–Y_10_—percentage of curcumin released after 2 h, 4 h, 6 h, 12 h, and 24 h (%).

The results obtained after fitting and calculation of the statistical parameters *R*^2^ and *Q*^2^, model validity and reproducibility, are shown in Figure 2. The values of *R*^2^ were greater than 0.8 for all responses and the values of *Q*^2^ were greater than 0.7 for all responses, except for Y_1_ (particle size) (*R*^2^ > 0.5), indicating that all responses were well fitted and predicted by the model. Furthermore, model validity and reproducibility values were greater than 0.7 and 0.6, respectively, suggesting a reduced experimental error.

The results of the ANOVA test (Table 4) showed that the *P*-value was lower than 0.05 for all responses and *P* for the lack of fit was greater than 0.05 for all responses, confirming that the proposed model is adequate, reliable, and has a good predictive power.

**Figure 3:**
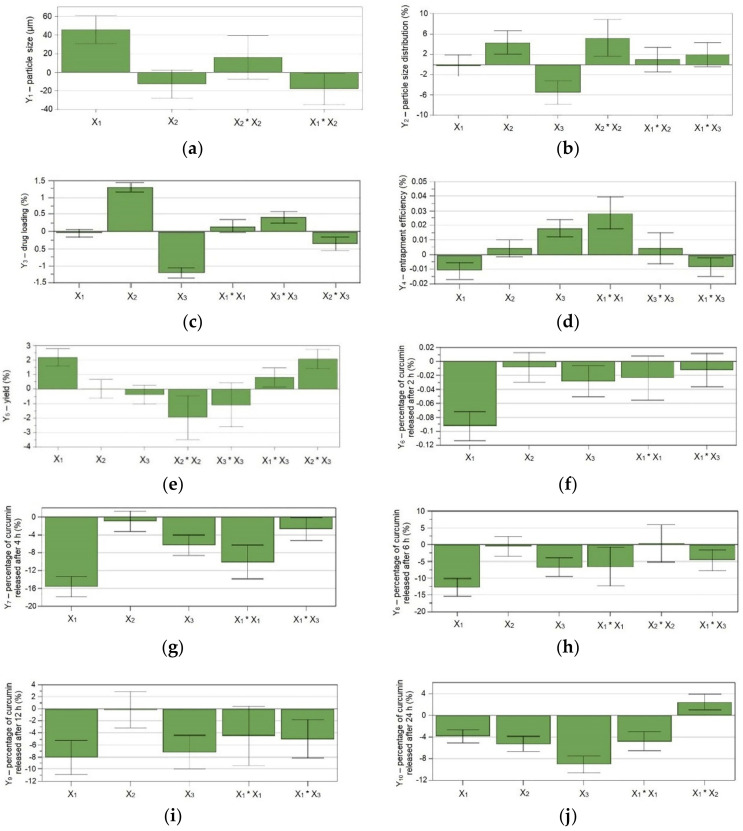
Regression coefficient plots showing the influence of formulation factors on the (**a**) particle size, (**b**) particle size distribution, (**c**) drug loading, (**d**) entrapment efficiency, (**e**) yield, and (**f**–**j**) percentage of curcumin released after 2 h, 4 h, 6 h, 12 h, and 24 h; X_1_—Eudragit^®^ FS proportion (%); X_2_—curcumin concentration (mg/mL); X_3_—concentration of the polymer mixture (mg/mL); Y_1_—particle size (μm); Y_2_—particle size distribution (%); Y_3_—drug loading (%); Y_4_—entrapment efficiency (%); Y_5_—yield (%); Y_6_–Y_10_—percentage of curcumin released after 2 h, 4 h, 6 h, 12 h, and 24 h (%).

**Figure 4:**
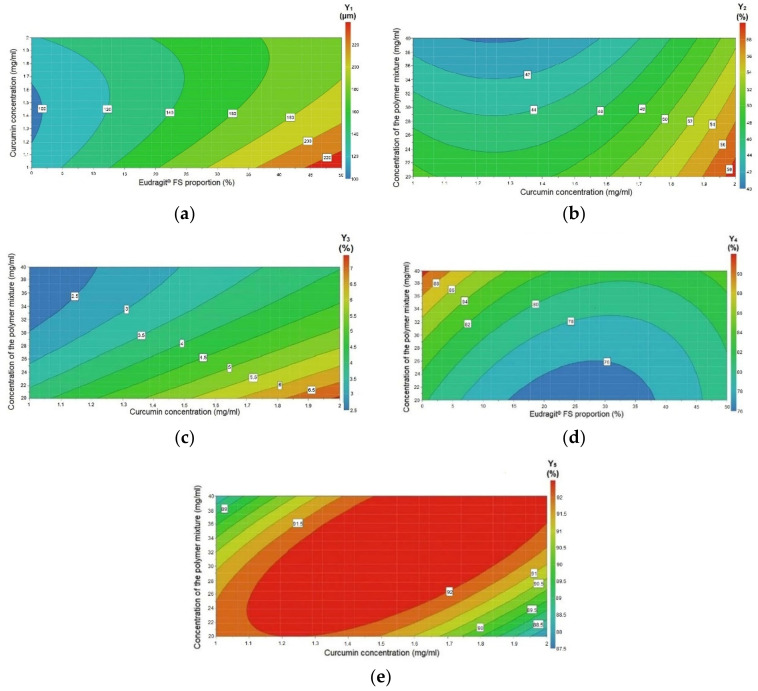
Contour plots showing the effect of the interaction between variables on the responses: (**a**) the effect of X_1_X_2_ on particle size; (**b**) the effect of X_2_X_3_ on particle size distribution; (**c**) the effect of X_2_X_3_ on drug loading; (**d**) the effect of X_1_X_3_ on entrapment efficiency; and (**e**) the effect of X_2_X_3_ on yield. X_1_—Eudragit^®^ FS proportion (%); X_2_—curcumin concentration (mg/mL); X_3_—concentration of the polymer mixture (mg/mL); Y_1_—particle size (μm); Y_2_—particle size distribution (%); Y_3_—drug loading (%); Y_4_—entrapment efficiency (%); Y_5_—yield (%).

**Figure 5:**
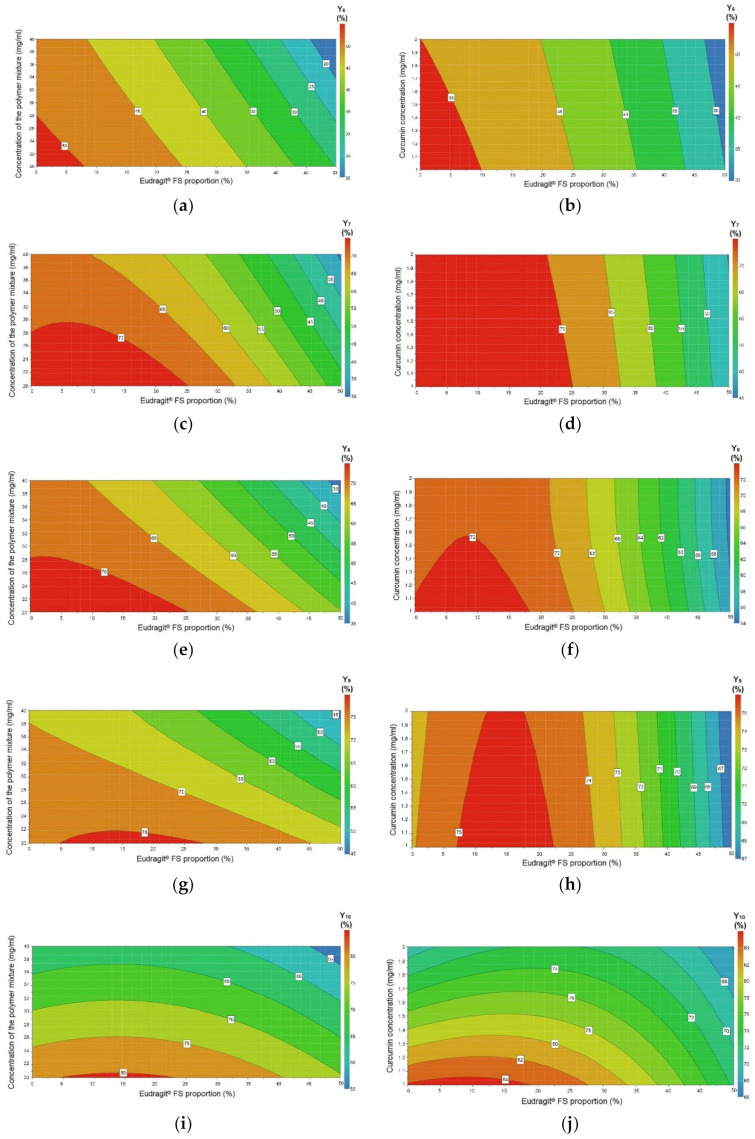
Contour plots showing the effect of the interaction between variables on the responses: (**a**,**c**,**e**,**g**,**i**) the effect of X_1_X_3_ on the percentage of curcumin released after 2 h, 4 h, 6 h, 12 h, and 24 h; and (**b**,**d**,**f**,**h**,**j**) the effect of X_1_X_2_ on the percentage of curcumin released after 2 h, 4 h, 6 h, 12 h, and 24 h. X_1_—Eudragit^®^ FS proportion (%); X_2_—curcumin concentration (mg/mL); X_3_—concentration of the polymer mixture (mg/mL); Y_6_–Y_10_—percentage of curcumin released after 2 h, 4 h, 6 h, 12 h, and 24 h (%).

The regression coefficient plots, shown in Figure 3, illustrate the influence of the formulation variables on the studied responses. Figure 4 and Figure 5 depict the contour plots, which illustrate the effect of the interaction between variables on the responses Y_1_–Y_5_ and Y_6_–Y_10_, respectively. The contour plots are generally generated for a better understanding of the main effects and the interactions between the investigated variables [25].

### 3.4. Effect of Formulation Factors on the Particle Size and Particle Size Distribution of Col-CUR-MPs

The particle size and particle size distribution are important characteristics of microparticles that directly influence their distribution and penetration in colitis tissues [2]. The particle size (Y_1_) of the 17 formulations ranged between 85.05 and 231.54 μm, while the particle size distribution varied in the range 40.30–60.60% (Table 3). The analysis of variance data for particle size and particle size distribution (Table 4) indicated a significant influence of the independent variables (*p* = 0.000 for particle size and *p* = 0.002 for particle size distribution) and the absence of lack of fit (*p* = 0.308 for particle size and *p* = 0.776 for particle size distribution).

Figure 3a showed that the Eudragit^®^ FS proportion (X_1_) influenced the size of the microspheres. Increasing the Eudragit^®^ FS proportion (X_1_) led to larger particles, while the interaction between the Eudragit^®^ FS proportion (X_1_) and the curcumin concentration (X_2_) determined a decrease in the size of the microspheres. The increase in the Eudragit^®^ FS proportion (X_1_) in the microparticles led to the increase of their diameter, suggesting that in the case of the microparticles that contained a polymeric mixture with a higher proportion of polycaprolactone, compact hydrophobic interactions occurred between curcumin and the more hydrophobic polymer, leading to microparticles with a smaller diameter [4]. The curcumin concentration (X_2_) did not influence the size of the microspheres. Even though the size of the microspheres usually increases with the increase in drug concentration [33], the drug–polymer ratio was probably not significant enough to influence the particles’ size. According to Figure 3b, the particle size distribution (Y_2_) was influenced by the curcumin and polymer concentrations. Increasing the curcumin concentration (X_2_) and reducing the polymer mixture concentration (X_3_) determined a higher particle size distribution.

The contour plot in Figure 4a also showed the influence of the Eudragit^®^ FS proportion (X_1_) and curcumin concentration (X_2_) on particle size. From the graphical representation, it can be observed that for a curcumin concentration value ranging between 1.4 and 1.5 mg/mL, the particle size significantly increased with increasing Eudragit^®^ FS proportion up to 40%. At an Eudragit^®^ FS proportion over 40%, the size of the microspheres continued to increase, but for lower curcumin concentration (1–1.2 mg/mL). Figure 4b illustrates the contour plot of the curcumin concentration (X_2_) and the concentration of the polymer mixture (X_3_) and theirs effect on particle size distribution, confirming the increase of the particle size distribution with a greater curcumin concentration and a lower polymer concentration.

### 3.5. Effect of Formulation Factors on the Curcumin Drug Loading and Entrapment Efficiency

The percentages of curcumin encapsulated in Col-CUR-MPs (Y_4_) were satisfying, ranging from 75.37% to 89.70% for all 17 formulations, and the drug loading (Y_3_) values varied between 2.02% and 7.33% (Table 3). The results of the ANOVA test for drug loading and entrapment efficiency (Table 4) indicated a significant influence of the independent variables on the responses (*p* = 0.000 for both drug loading and entrapment efficiency) and absence of lack of fit (*p* = 0.800 for drug loading and *p* = 0.907 for entrapment efficiency).

According to Figure 3c, the curcumin drug loading (Y_3_) was influenced by the curcumin concentration (X_2_) and the concentration of the polymer mixture (X_3_). Increasing the curcumin concentration (X_2_) and reducing the concentration of the polymer mixture (X_3_) determined a higher drug loading. The curcumin entrapment efficiency (Y_4_) appeared to be influenced by the Eudragit^®^ FS proportion (X_1_) and the concentration of the polymer mixture (X_3_), according to Figure 3d. Increasing the Eudragit^®^ FS proportion (X_1_) reduced the curcumin entrapment efficiency, while increasing the concentration of the polymer mixture (X_3_) increased the curcumin entrapment efficiency. Based on scientific literature information, drug entrapment within micro- and nanospherical systems increases with polymer concentration increase, which can be explained by the delay of the drug diffusion from the polymeric solution with high viscosity determined by a high polymer concentration [40].

Figure 4c, which shows the variables’ interactions, revealed that the curcumin and polymer mixture concentrations had an antagonistic effect on the curcumin drug loading. Similarly, the Eudragit^®^ FS proportion and the polymer mixture concentration showed an antagonistic effect on the curcumin entrapment efficiency (Figure 4d).

### 3.6. Effect of Formulation Factors on the Yield of Col-CUR-MPs

The percentages of Col-CUR-MPs yield (Y_5_) were satisfying, ranging from 82.95% to 93.83% (Table 3). The statistical analysis of the data for the yield (Table 4) also indicated the significant influence which the independent variables exhibited (*p* = 0.000) and the absence of lack of fit (*p* = 0.795).

Figure 3e showed that the Eudragit^®^ FS proportion (X_1_) influenced the yield: increasing the Eudragit^®^ FS proportion (X_1_) led to a higher yield. This finding contradicts the results of other studies, in which the researchers observed that an increase in the copolymer concentration (Eudragit^®^ S 100) affected the percentage yield, due to the increase in the viscosity of the solution [41]. The contour plot in Figure 4e illustrates that there is a specific range for curcumin and polymer mixture concentrations, for which the percentage yield of the obtained microspheres was the highest: 1.2–1.7 mg/mL and 26–36 mg/mL, respectively. Outside these intervals, the percentage yield decreased.

### 3.7. Effect of Formulation Factors on the In Vitro Release of Curcumin

Despite the fact that curcumin is an important anti-inflammatory agent for the treatment of IBD, its therapeutic efficiency after oral administration is relatively low. To overcome this issue, researchers have sought to develop strategies for colon-targeted curcumin delivery and sustained drug release systems. In our study, the in vitro release profiles of curcumin from microspheres were studied in media with a pH-simulating GIT environment, as shown in Figure 1 [4]. The percentages of curcumin released after 2 h in acidic environment (Y_6_) widely ranged between 14.50% and 52.74%, all formulations exceeding the limit of 10% required for colonic release formulations [37]; however, N6 (18.78%), N8 (14.50%), and N10 (19.54%) formulations were closer to the proposed goal. After changing the dissolution medium to one with pH 6.8 in order to simulate the pH of the intestinal environment, the amount released further increased, and the percentages released after 24 h were within the range 49.92% and 80.32%. The results of the ANOVA test for the percentages of curcumin released at different time intervals (Table 4) indicated a significant influence of the independent variables on the responses (*p* = 0.000 for all studied time frames) and absence of lack of fit (0.450 < *p* < 0.767).

The influence of the factors on the in vitro release of curcumin is illustrated in Figure 3f–j. The percentages of curcumin released in pH 1.2 (Y_6_) decreased with the increase of the Eudragit^®^ FS proportion (X_1_) and the concentration of the polymer mixture (X_3_) (Figure 3f). The percentages of curcumin released in pH 6.8 (Y_7_–Y_10_) also decreased with the increase of the Eudragit^®^ FS proportion (X_1_), but the influence of the enterosoluble polymer was less important over time (Figure 3g–j). The other factor which influenced the in vitro release of curcumin in pH 6.8 was the concentration of the polymer mixture (X_3_); increasing the polymer mixture concentration determined a reduction in curcumin release. The curcumin concentration (X_2_) influenced the in vitro release of curcumin only after 24 h; its influence was also a negative one, namely increasing the curcumin concentration determined a reduction in curcumin release. The published data regarding the influence of formulation variables on drug release state that the release mechanism can easily be modulated by the weight of the used polymers: higher molecular weight polymers show slower release of the drugs. According to the literature, the higher viscosity polymers require higher energy to pull the chain from their matrix. Another factor contributing to a slower drug release is the formation of a thicker gel layer after hydration [40]. This explains the results obtained in our study, namely that an increase of the higher molecular weight polymer, Eudragit^®^ FS (280,000 g/mol), determined a slower release of curcumin when compared to polymer mixtures containing a higher proportion of polycaprolactone (45,000 g/mol). Eudragit^®^ FS is also a methacrylic copolymer capable of targeted transport to the colon by dissolving at pH 7.0 by the formation of salts [16]; therefore, it was expected to decrease the release of curcumin. The decrease in the percentages of curcumin released with increasing concentrations of the polymer mixture can be explained by the same theory mentioned before regarding the entrapment efficiency. A high concentration of the polymer may cause the formation of a thicker, more viscous gel layer after hydration, which delays drug diffusion and slows its release.

The contour plots in Figure 5 show the interaction between variables and their effect on the responses: the effect of X_1_X_3_ on the percentage of curcumin released after 2 h, 4 h, 6 h, 12 h, and 24 h ((a), (c), (e), (g), (i)); and the effect of X_1_X_2_ on the percentage of curcumin released after 2 h, 4 h, 6 h, 12 h and 24 h ((b), (d), (f), (h), (j)). For all the studied in vitro release time intervals of curcumin from microspheres, the Eudragit^®^ FS proportion (X_1_) and the concentration of the polymer mixture (X_3_) showed a synergistic effect (Figure 5a,c,e,g,i). Regarding the interaction between the Eudragit^®^ FS proportion (X_1_) and curcumin concentration (X_2_) (Figure 5b,d,f), the drug release variation at 2 h, 4 h, and 6 h was significant when increasing the Eudragit^®^ FS proportion, but rather moderate by modifying the curcumin concentration. However, at 12 h and 24 h (Figure 5h,j), the curcumin concentration impact increased and the two variables presented a synergistic effect.

### 3.8. Establishment of the Design Space and Validation of the Model

The design space delineates the relationship between the material attributes/process parameters and CQAs and is determined from the common region of successful operating ranges for multiple CQAs [23]. The wider the design space, the more robust and flexible the process is to adjust variations.

**Figure 6:**
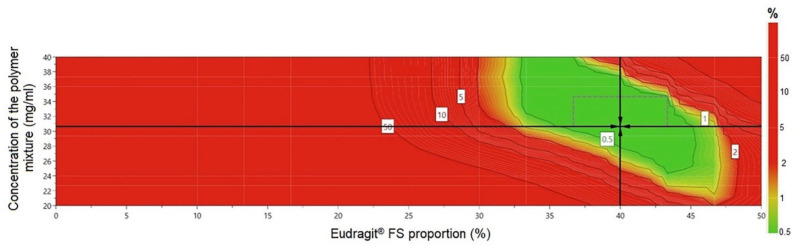
Design space for the formulation of Col-CUR-MPs, for a curcumin concentration set at 1.2 mg/mL, represented as a function of Eudragit^®^ FS proportion and the concentration of the polymer mixture.

In this study, the design space was constructed for a curcumin concentration set at 1.2 mg/mL, taking into account the key parameters that have been demonstrated to affect product quality the most: Eudragit^®^ FS proportion (X_1_) and concentration of the polymer mixture (X_3_), as illustrated in Figure 6. This figure shows a green region, which corresponds to the optimal design space region with feasible response, and a red region, denoting the area where response did not fit the desired product criteria. The criterion for selecting the optimized region was to attain lower drug release in the stomach and proximal parts of the intestine and higher drug release in the colon. The robust setpoint formulation was prepared in the following conditions, according to the plots indicated by the design space (Figure 6): the Eudragit^®^ FS proportion (X_1_) was set at 40% and the concentration of the polymer mixture (X_3_) was set at 30.66 mg/mL. The preparation conditions for the formulation outside the design space were: Eudragit^®^ FS proportion (X_1_)—26%, and the concentration of the polymer mixture (X_3_)—35 mg/mL. Both formulations were prepared in triplicate and were further analyzed and characterized for particle size, particle size distribution, drug loading, entrapment efficiency, yield, and percentages of drug released at different time intervals during 24 h.

The process robustness and accuracy of the obtained model were further evaluated by performing additional tests. The predicted and average experimental results obtained in the optimization study for the formulations within and outside the design space are illustrated in Table 5. The yield (Y_5_) and percentage of curcumin released after 24 h (Y_10_) were not included as criteria in the optimization plan, but the values obtained were satisfactory: 88.69% ± 2.62% for the robust setpoint formulation and 81.19% ± 4.13% for the formulation outside the design space, and 45.66% ± 3.73% for the robust setpoint formulation and 51.03 ± 3.05% for the formulation outside the design space, respectively.

The robust setpoint formulation was found to possess good characteristics. The particle size and particle size distribution were satisfactory (171.25 ± 4.84 μm and 47.58% ± 8.11%, respectively), and the drug loading and drug entrapment were high (3.69% ± 0.24% and 98.26% ± 6.38%, respectively). Regarding the in vitro release of the drug from the microspheres, the robust setpoint formulation was appropriate for a colonic drug delivery system, as the percentage values obtained were gradual: 20.41% ± 10.63% after 2 h at pH 1.2 (Y_6_), 35.56% ± 7.61% after 4 h (Y_7_), 40.52% ± 6.79% after 6 h (Y_8_), and 44.93% ± 5.70% after 12 h (Y_9_) at pH 6.8. During the transit of a colon-specific drug delivery system through the stomach and small intestine, which usually corresponds to 5 h, low percentages of released curcumin, as the ones obtained for the robust setpoint formulation, represent an advantage. After 5–6 h spent in the GIT, the colon-specific drug delivery system is usually localized in the colon, where a complete release of the drug is desired. However, the more gradual release obtained for the robust setpoint formulation proved a better achievement of a colonic release. All the experimental results were within the limits of the predicted range, except for the entrapment efficiency. The bias between the experimental and predicted values was high for some responses; however, the experimental data were satisfactory for a colon drug delivery system.

For the formulation outside the design space, except for the particle size distribution, all the experimental results were outside the limits of the predicted range, which indicates a poor correlation between the experimental results and the predicted ones.

The above presented results show the following: the particle size is lower for the robust setpoint formulation than for the formulation outside the design space; the particle size distribution, drug loading, and entrapment efficiency were higher for the robust setpoint formulation than for the formulation outside the design space. The percentages of curcumin released in the gastric and intestinal simulated environments were also lower for the robust setpoint formulation than for the formulation outside the design space for all the studied time intervals. This proves that a higher percentage of enterosoluble polymer, Eudragit^®^ FS, led to microspheres which allowed a slower release of curcumin than the microspheres prepared with a lower percentage of enterosoluble polymer. Therefore, the robust setpoint formulation confirms the validity of the design space, by the fact that working inside the design space will lead to predictable results, whereas working outside the design space will lead to results that cannot be accurately predicted.

### 3.9. In Vivo Release of FITC from Microspheres

**Figure 7:**
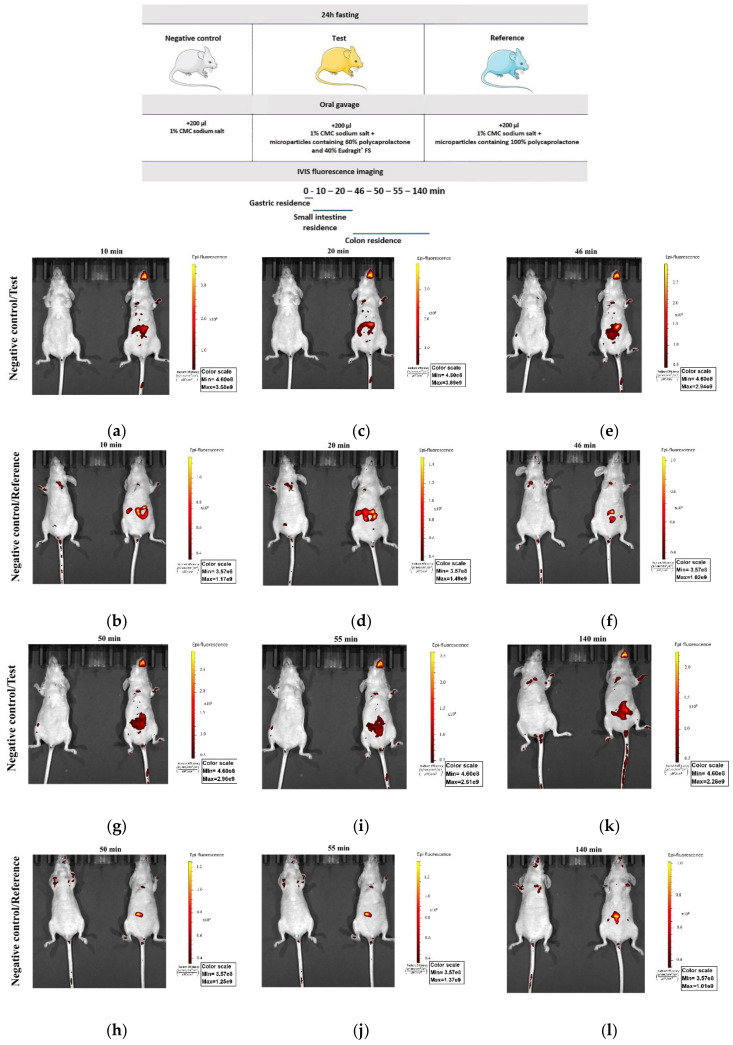
Fluorescein isothiocyanate (FITC) distribution in the gastrointestinal tract (GIT) (negative control group—left, test group or reference group—right): (**a**) 10 min, test; (**b**) 10 min, reference; (**c**) 20 min, test; (**d**) 20 min, reference; (**e**) 46 min, test; (**f**) 46 min, reference; (**g**) 50 min, test; (**h**) 50 min, reference; (**i**) 55 min, test; (**j**) 55 min, reference; (**k**) 140 min, test; (**l**) 140 min, reference.

The in vivo FITC release from the microspheres in mice was studied by IVIS in order to determine the residence time of the colon-specific drug delivery system in different parts of the GIT. Figure 7a,c,e,g,i,k shows the in vivo images of FITC distribution at different times (10, 20, 46, 50, 55, and 140 min) in mice after the administration of an aqueous solution of 1% carboxymethylcellulose sodium salt (negative control group, left), and microparticles containing a mixture of polycaprolactone and Eudragit^®^ FS (60:40, *w/w*) suspended in 200 μL aqueous solution of 1% carboxymethylcellulose sodium salt (test group, right). The test formulation corresponds to the robust setpoint’s composition established through QbD, namely 40% Eudragit^®^ FS proportion and 60% polycaprolactone.

Figure 7b,d,f,h,j,l shows the in vivo images of FITC distribution at different times (10, 20, 46, 50, 55, and 140 min) in mice after the administration of an aqueous solution of 1% carboxymethylcellulose sodium salt (negative control group, left), and microparticles containing 100% polycaprolactone suspended in 200 μL aqueous solution of 1% carboxymethylcellulose sodium salt (reference group, right). The predetermined time points were chosen according to Chen et al.; their findings showed that the gastric residence time in mice is 2 min, the small intestinal residence time is 42 min, and after 44 min, the microspheres enter the colon physiological environment [26].

The negative control group did not present fluorescence for the experiment period (from 10 to 140 min). As for the test group, a fluorescent spot was found on the stomach of mice at 10 min after administration (Figure 7a). At 20 min after administration, the fluorescence spot moved from the stomach to the small intestine (Figure 7c), and at 46 min after administration the fluorescence spot reached the colon (Figure 7e). At 50 and 55 min after intragastric administration, it can be seen that the fluorescence spread in the colon (Figure 7g,i), and the intensity decreased due to the metabolism of FITC. At 140 min after intragastric administration (Figure 7k), the fluorescence spot moved further in the colon and its intensity increased, probably due to the release of a new amount of FITC. After the intragastric administration of the reference formulation, the in vivo images showed a fluorescent spot in the stomach of mice at 10 min after administration (Figure 7b) and the fluorescence advancing in the small intestine at 20 min after administration (Figure 7d). From 46 to 140 min after intragastric administration (Figure 7f,h,j,l), it can be seen that the fluorescence decreased greatly, which can be explained by the disaggregation of the FITC-loaded microparticles in the stomach and, therefore, the absorption of FITC. The absorbed FITC did not have a sufficiently intense fluorescence to be detected after release from the system and spreading throughout the body through the vascular system.

**Figure 8:**
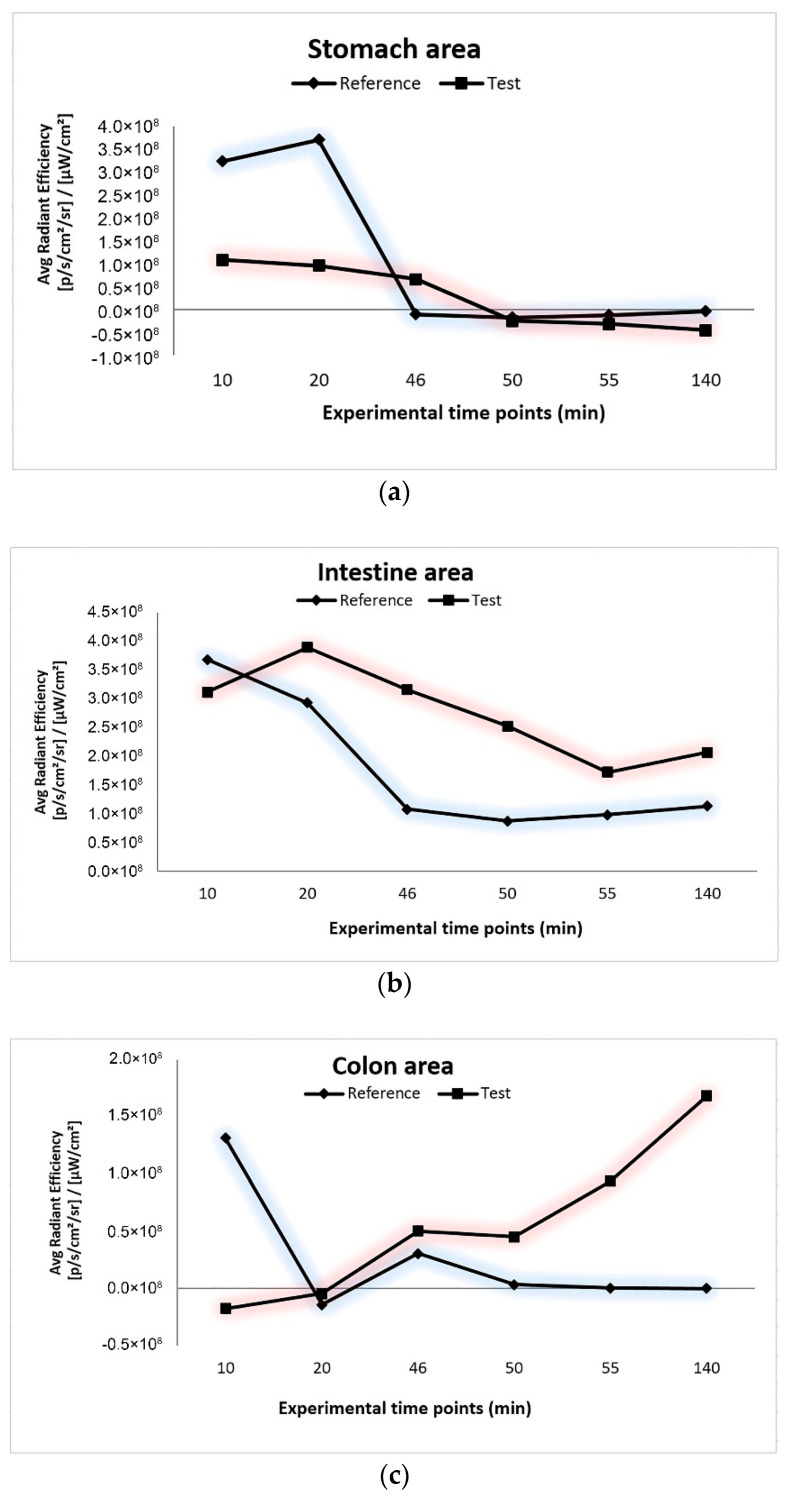
Average radiant efficiency (p/s/cm^2^/sr)/(μW/cm^2^) of in vivo imaging system (IVIS) imaged mice: (**a**) stomach area; (**b**) intestine area; (**c**) colon area.

In order to perform a semi-quantitative fluorescence distribution analysis in the three areas of the GIT (stomach, small intestine, and colon), the graphical representation of the average radiant efficiency versus the experimental time points for the three areas was obtained: stomach (Figure 8a), intestine (Figure 8b), and colon (Figure 8c). The reference group showed a maximum average radiant efficiency after 10–20 min after intragastric administration for all three areas; therefore, there was a significant release of FITC in the stomach and small intestine. After 46 min (the moment when the system reaches the colon), the average radiant efficiency was close to 0, which proves that all FITC had already been released. The test group presented very low values of average radiant efficiency in the stomach compared to the reference group (Figure 8a), proving the ability of the system to prevent drug release in this part of the GIT. Figure 8c illustrated a negligible release of FITC from the test formulation in the first 20 min and an important release from 46 min, demonstrating that the test formulation has a good ability to prevent the release of the encapsulated agent in the superior parts of the GIT and to assure its targeted release in the colon.

Therefore, using the QbD approach, we were able to identify the test formulation and determine its in vivo release behavior compared to the reference formulation. Our results showed that the reference formulation did not reveal colon-targeting abilities, with the absorption of FITC occurring in the stomach and the upper GIT. Instead, the test formulation managed to deliver and release the active compound to the site of action, as the in vivo images and the semi-quantitative analysis illustrated a significant fluorescence in the colon.

## 4. Conclusions

In this study, curcumin microspheres for colon-specific delivery were successfully formulated, prepared, characterized, and optimized using QbD principles. The Eudragit^®^ FS proportion, curcumin concentration, and concentration of the polymer mixture were identified as critical parameters affecting the CQAs of the microspheres: particle size, particle size distribution, drug loading, entrapment efficiency of curcumin, and the percentages of drug released in media simulating the GIT environment. In order to obtain a predictive model by performing a small number of experiments, the central composite face experimental design was found to be appropriate. Following the analysis of the experimental design, the design space was established in order to formulate microspheres with the desired QTPP. The optimum formulation, corresponding to the robust setpoint, was characterized and found to possess good characteristics: high drug entrapment and gradual release of the drug from the microspheres, appropriate for a colonic drug delivery system.

Furthermore, the formulation developed by the QbD approach, corresponding to the robust setpoint, was evaluated in vivo by the IVIS imaging system for the release of fluorescein in different parts of the GIT. Our results showed that microparticles containing a mixture of polycaprolactone and Eudragit^®^ FS (60:40, *w/w*) had a well-controlled release behavior and the ability to effectively deliver active compounds into the colon.

In conclusion, the development of the colon-specific delivery microspheres with the desired QTPP using QbD demonstrated the great value of this approach: identifying the critical variables influencing the manufacturing process of microspheres, understanding the influence of formulation and process variables on the CQAs, and determining the optimum conditions for developing Col-CUR-MPs. Due to the fact that the Col-CUR-MPs show great potential as a colon-specific drug delivery system, it would be justified to further study this formulation in vivo in animal models with experimentally induced colitis in order to evaluate the reliability and anti-inflammatory effect of this colon drug delivery system.

## Figures and Tables

**Table 1:** Independent variables and their levels.

Independent Variable	Symbol	Level		
		−1	0	+1
Eudragit^®^ FS proportion (%)	X_1_	0	25	50
Curcumin concentration (mg/mL)	X_2_	1	1.5	2
Concentration of the polymer mixture (mg/mL)	X_3_	20	30	40

**Table 2:** Experimental design matrix.

Exp. ^(1)^ No.	Exp. ^(1)^ Name	Run Order	X_1_^(2)^	X_2_^(3)^	X_3_^(4)^
1	N1	13	0	1	20
2	N2	12	50	1	20
3	N3	10	0	2	20
4	N4	11	50	2	20
5	N5	2	0	1	40
6	N6	1	50	1	40
7	N7	14	0	2	40
8	N8	3	50	2	40
9	N9	17	0	1.5	30
10	N10	15	50	1.5	30
11	N11	7	25	1	30
12	N12	4	25	2	30
13	N13	9	25	1.5	20
14	N14	6	25	1.5	40
15	N15	16	25	1.5	30
16	N16	5	25	1.5	30
17	N17	8	25	1.5	30

^(1)^ Experiment; ^(2)^ Eudragit^®^ FS proportion (%); ^(3)^ curcumin concentration (mg/mL); ^(4)^ concentration of the polymer mixture (mg/mL).

**Table 3:** Responses matrix for particle size (μm) (Y_1_), particle size distribution (%) (Y_2_), drug loading (%) (Y_3_), entrapment efficiency (%) (Y_4_), yield (%) (Y_5_), and percentages of curcumin released after 2 h, 4 h, 6 h, 12 h, and 24 h (%) (Y_6_–Y_10_).

Exp. ^(1)^ No.	Exp. ^(1)^ Name	Y_1_	Y_2_	Y_3_	Y_4_	Y_5_	Y_6_	Y_7_	Y_8_	Y_9_	Y_10_
1	N1	85.05	54.70	3.84	80.05	89.59	52.74 ± 3.9	72.83 ±1.6	72.76 ± 2.4	73.70 ± 4.8	70.72 ± 4.0
2	N2	228.50	48.10	3.80	79.65	91.24	22.96 ± 1.2	43.72 ±7.5	52.59 ± 11.6	64.95 ± 2.7	63.89 ± 1.3
3	N3	108.22	60.60	7.33	80.58	84.57	50.09 ± 6.2	71.14 ±2.3	70.09 ± 1.3	72.29 ± 1.8	70.02 ± 3.0
4	N4	205.57	56.70	7.29	80.11	87.87	27.63 ± 1.0	49.06 ±1.1	57.99 ± 1.6	67.68 ± 1.2	65.82 ± 0.5
5	N5	125.73	40.30	2.19	89.70	82.95	49.06 ± 8.8	66.28 ± 10.0	67.47 ± 9.5	67.74 ± 8.7	67.27 ± 8.0
6	N6	231.54	40.60	2.02	82.32	87.86	18.78 ± 0.2	34.05 ± 3.8	37.64 ± 0.9	47.12 ± 8.3	54.32 ± 10.1
7	N7	137.06	47.00	4.05	84.95	86.28	48.94 ± 6.1	67.25 ± 3.0	69.86 ± 2.5	74.27 ± 2.0	72.82 ± 0.5
8	N8	146.90	52.10	4.09	85.15	92.82	14.50 ± 2.0	26.43 ± 2.8	30.36 ± 5.0	41.59 ± 18.9	49.92 ± 18.7
9	N9	103.58	41.70	4.07	85.49	88.28	46.04 ± 3.9	68.24 ± 6.5	68.66 ± 8.4	70.93 ± 3.5	68.59 ± 4.5
10	N10	206.13	44.10	3.77	79.23	93.83	19.54 ± 1.0	35.99 ± 7.8	41.84 ± 8.9	56.98 ± 14.1	61.51 ± 9.3
11	N11	187.03	58.90	2.47	76.37	93.62	44.00 ± 2.5	66.63 ± 2.6	70.96 ± 3.1	77.38 ± 10.0	73.86 ± 6.0
12	N12	135.26	56.90	4.94	78.98	89.30	41.36 ± 4.2	59.80 ± 7.5	60.18 ± 11.3	64.28 ± 17.2	62.90 ± 16.8
13	N13	111.05	54.70	5.41	77.46	92.95	50.11 ± 2.5	73.80 ± 3.0	73.18 ± 6.2	79.59 ± 1.8	80.32 ± 0.2
14	N14	164.00	58.00	2.93	80.86	89.75	29.98 ± 14.8	53.28 ± 19.4	53.37 ± 20.8	55.43 ± 23.5	53.71 ± 21.9
15	N15	152.19	48.90	3.71	77.75	91.48	37.95 ± 0.0	55.63 ± 7.3	59.02 ± 7.9	59.89 ± 9.9	58.64 ± 9.4
16	N16	123.85	41.50	3.59	75.37	88.50	46.19 ± 0.6	62.60 ± 9.9	65.60 ± 7.5	68.96 ± 10.1	69.75 ± 7.3
17	N17	146.52	49.10	3.89	81.79	90.04	39.72 ± 2.4	65.58 ± 2.1	67.84 ± 0.4	72.65 ± 2.2	71.01 ± 1.6

^(1)^ Experiment.

**Table 4:** Statistical parameters *R^2^* and *Q^2^*, and analysis of variance (ANOVA) showing fitting of the experimental data to the model.

Response	*R* ^2 (1)^	*Q* ^2 (2)^	DF ^(3)^	SS ^(4)^	MS ^(5)^	F ^(6)^	*p*-Value ^(7)^	Lack of Fit	Model Validity
Y_1_^(8)^	0.821	0.590	12	5703.86	475.322	13.790	0.000	0.308	0.705
Y_2_^(9)^	0.889	0.739	8	69.721	8.715	10.723	0.002	0.776	0.937
Y_3_^(10)^	0.990	0.959	7	0.096	0.014	111.627	0.000	0.800	0.944
Y_4_^(11)^	0.926	0.786	8	0.0004	0.00005	16.758	0.000	0.907	0.975
Y_5_^(12)^	0.970	0.803	6	3.485	0.581	28.142	0.000	0.795	0.942
Y_6_^(13)^	0.937	0.871	9	0.006	0.0007	26.827	0.000	0.767	0.933
Y_7_^(14)^	0.968	0.900	10	108.119	10.812	61.085	0.000	0.450	0.800
Y_8_^(15)^	0.949	0.832	9	130.142	14.460	28.041	0.000	0.745	0.926
Y_9_^(16)^	0.911	0.733	9	140.215	15.579	18.421	0.000	0.459	0.805
Y_10_^(17)^	0.985	0.931	7	11.762	1.680	89.003	0.000	0.495	0.824

^(1)^ Regression coefficient; ^(2)^ predictive power of the model; ^(3)^ degrees of freedom; ^(4)^ sum of squares; ^(5)^ mean of square (variance); ^(6)^ Fisher’s ratio; ^(7)^ probability; ^(8)^ particle size (μm); ^(9)^ particle size distribution (%); ^(10)^ drug loading (%); ^(11)^ entrapment efficiency (%); ^(12)^ yield (%); ^(13–17)^ percentage of curcumin released after 2 h, 4 h, 6 h, 12 h, and 24 h (%).

**Table 5:** Predicted and experimental results for the formulation within the design space (robust setpoint) and the formulation outside the design space.

CQA ^(1)^	Predicted Results	Predicted Range	Experimental Results	Bias (%)
Formulation within the design space				
Y_1_^(2)^	190.20	96.64–235.23	171.25 ± 4.84	−9.96
Y_2_^(3)^	43.55	37.27–61.50	47.58 ± 8.11	9.25
Y_3_^(4)^	2.92	2.00–7.21	3.69 ± 0.24	26.37
Y_4_^(5)^	77.72	74.23–91.83	98.26 ± 6.38	26.43
Y_6_^(6)^	31.75	13.08–52.23	20.41 ± 10.63	−35.72
Y_7_^(7)^	50.64	27.81–75.05	35.56 ± 7.61	−29.78
Y_8_^(8)^	53.02	32.11–73.56	40.52 ± 6.79	−23.58
Y_9_^(9)^	61.19	43.29–76.05	44.93 ± 5.70	−26.57
Formulation outside the design space				
Y_1_^(2)^	158.53	142.94–174.12	180.51 ± 6.81	13.86
Y_2_^(3)^	41.58	38.77–44.39	43.08 ± 5.04	3.61
Y_3_^(4)^	2.56	2.40–2.72	3.11 ± 0.22	21.48
Y_4_^(5)^	78.60	77.24–79.98	93.80 ± 6.67	19.34
Y_6_^(6)^	38.92	34.89–42.71	27.33 ± 2.47	−29.78
Y_7_^(7)^	60.35	56.85–63.84	46.21 ± 7.29	−23.43
Y_8_^(8)^	59.73	55.12–64.34	46.49 ± 3.78	−22.17
Y_9_^(9)^	64.29	59.43–69.16	50.45 ± 3.01	−21.53

^(1)^ Critical quality attribute; ^(2)^ particle size (%); ^(3)^ particle size distribution (%); ^(4)^ drug loading (%); ^(5)^ entrapment efficiency (%); ^(6–9)^ percentage of curcumin released after 2 h, 4 h, 6 h, and 12 h (%).
